# Mitochondrial and nuclear genetic analyses of the tropical black-lip rock oyster (*Saccostrea echinata*) reveals population subdivision and informs sustainable aquaculture development

**DOI:** 10.1186/s12864-019-6052-z

**Published:** 2019-09-12

**Authors:** Samantha J. Nowland, Catarina N. S. Silva, Paul C. Southgate, Jan M. Strugnell

**Affiliations:** 1 Aquaculture Unit, Department of Primary Industry and Resources, Northern Territory Government, GPO Box 3000, Darwin, NT 0801 Australia; 2 School of Science and Engineering, University of the Sunshine Coast, 90 Sippy Downs Drive, Sippy Downs, Queensland 4556 Australia; 3 Centre for Sustainable Tropical Fisheries and Aquaculture, and College of Science and Engineering, James Cook University, Townsville, Queensland 4811 Australia; 4 Australian Centre for Pacific Islands Research and School of Science and Engineering, University of the Sunshine Coast, Maroochydore, Queensland 4556 Australia

**Keywords:** Population genetics, Aquaculture, Black-lip rock oyster, Genetic management, *Saccostrea*

## Abstract

**Background:**

The black-lip rock oyster (*Saccostrea echinata*) has considerable potential for aquaculture throughout the tropics. Previous attempts to farm *S. echinata* failed due to an insufficient supply of wild spat; however, the prospect of hatchery-based aquaculture has stimulated renewed interest, and small-scale farming is underway across northern Australia and in New Caledonia. The absence of knowledge surrounding the population genetic structure of this species has raised concerns about the genetic impacts of this emerging aquaculture industry. This study is the first to examine population genetics of *S. echinata* and employs both mitochondrial cytochrome c oxidase subunit I gene (COI) and single nucleotide polymorphism (SNP) markers.

**Results:**

The mitochondrial COI data set included 273 sequences of 594 base pair length, which comprised 74 haplotypes. The SNP data set included 27,887 filtered SNPs for 272 oysters and of these 31 SNPs were identified as candidate adaptive loci. Data from the mitochondrial COI analyses, supports a broad tropical Indo-Pacific distribution of *S. echinata,* and showed high haplotype and nucleotide diversities (0.887–1.000 and 0.005–0.008, respectively). Mitochondrial COI analyses also revealed a ‘star-like’ haplotype network, and significant and negative neutrality tests (Tajima’s *D =* − 2.030, Fu’s *F*s = − 25.638, *P* < 0.001) support a recent population expansion after a bottleneck. The SNP analyses showed significant levels of population subdivision and four genetic clusters were identified: (1) the Noumea (New Caledonia) sample location; (2) the Bowen (north Queensland, Australia) sample location, and remaining sample locations in the Northern Territory, Australia (*n* = 8) were differentiated into two genetic clusters. These occurred at either side of the Wessel Islands and were termed (3) ‘west’ and (4) ‘east’ clusters, and two migrant individuals were detected between them. The SNP data showed a significant positive correlation between genetic and geographic distance (Mantel test, *P < 0.001*, *R*^*2*^ = 0.798) and supported isolation by distance. Three candidate adaptive SNPs were identified as occurring within known genes and gene ontology was well described for the sex peptide receptor gene.

**Conclusions:**

Data supports the existence of genetically distinct populations of *S. echinata*, suggesting that management of wild and farmed stocks should be based upon multiple management units. This research has made information on population genetic structure and connectivity available for a new aquaculture species.

**Electronic supplementary material:**

The online version of this article (10.1186/s12864-019-6052-z) contains supplementary material, which is available to authorized users.

## Background

For farmed marine species, understanding population structure and how populations respond to the environment can inform broodstock selection, translocation boundaries, and aquaculture induced evolution [1, 2]. Potential impacts from aquaculture relate to the introgression of exotic alleles, caused by processes such as: interbreeding or outbreeding of wild and cultivated populations, the outcompeting of native alleles by introduced alleles, and translocations, all of which can negatively alter the genetic diversity of remnant populations [3]. Therefore, an understanding of natural population genetics, before aquaculture occurs, is necessary for the proactive management of these impacts [4, 5]. Understanding the genetics of natural populations also provides potential benefits for improving aquaculture and biosecurity practices as many adaptive traits in the wild are important production traits, such as; growth rate, environmental tolerances, and disease resistance [6, 7].

Many methods exist to investigate population genetics, and those such as mitochondrial genes provide useful tools for identifying and improving our understanding of relationships among species [8, 9]. More sophisticated and increasingly affordable methods are becoming available for application on non-model species; which often lack genomic resources and includes many species used for aquaculture [10]. The advent of next generation sequencing technologies and restriction-site associated DNA (RADseq) genotyping methods, has enabled delivery of genome-wide single nucleotide polymorphisms (SNPs) for any organism (e.g. non-model species), at reasonable costs [11, 12]. Genome-wide SNPs can reveal fine-scale patterns of population structure, dispersal capabilities, population assignment, and detect signatures of selection [13–15], with much higher resolution than traditional markers (e.g. allozymes and microsatellites). The use of these technologies in the detection of fine-scale structure and signatures of selection are important for determining management units, particularly in populations that may otherwise appear homogenous in their distribution [10].

Marine bivalves are characterised by large population sizes, high fecundity, and planktonic larvae with considerable dispersal potential [14, 16, 17]. In sessile bivalve populations, such as oysters, gene flow is achieved through the dispersal of gametes which frequently results in high genetic diversity and weak genetic differentiation [18]. This is true for the tropical oyster *Crassostrea corteziensis* in the Gulf of California where allozyme analyses revealed a single panmictic population, possessing high genetic diversity [19]. However, deviations from this trend can occur, usually as a result of larval behaviour, environmental gradients, anthropogenic effects, and physical barriers [20–22]. For example, analyses of mitochondrial and SNP data revealed many subpopulations of *Crassostrea virginica* throughout the Gulf of Mexico, despite its potential for high gene flow [23]. Similar results of population differentiation in a species with high dispersal capabilities, have been reported for *Crassostrea iredalei* throughout Malaysia [24] and *Ostrea edulis* along the European coast [25]. Therefore, without detailed population genetic analyses, predictions cannot be made regarding the extent of population genetic structure of oyster species.

The black-lip rock oyster, *Saccostrea echinata* (Quoy and Gaimard, 1835), is a tropical marine bivalve that is reported to have broad geographic distribution throughout the Indo-Pacific [26]. Research is currently underway to improve the commercial aquaculture potential of *S. echinata* due to the success of small-scale farms (< 1 t per annum) in northern Australia, eastern Australia, and New Caledonia (JR Collison pers. comm. 2018). Production based on wild seed supply has been a bottleneck for the industry [27], and this is still the case. Development of hatchery protocols for this species is therefore considered a prerequisite for advancement and progress to commercial scale hatchery production is being made [28, 29]. Larvae of *S. echinata* are planktonic and development occurs over approximately 21 days in the hatchery, after which metamorphosis and settlement takes place [29]. Larvae may conceivably travel hundreds of kilometres before settlement, which leads to the hypothesis that wild populations possess low levels of genetic structure [30]. However, studies on related oyster species have reported both panmictic [19, 30] and highly divergent [23, 24] population structures. To achieve the genetic conservation of oyster stocks in the face of developing aquaculture efforts, a sound knowledge of population genetic structure is essential. A genetic stock assessment of natural *S. echinata* populations, before commencement of commercial-scale aquaculture, would provide biological information upon which responsible management and hatchery production could be based. This study aims to fill this knowledge gap by analysing both mitochondrial and nuclear DNA markers to assess the population genetic structure and the neutral and adaptive genetic diversity of *S. echinata* across northern Australia.

## Results

### Mitochondrial COI data

**Fig. 1:**
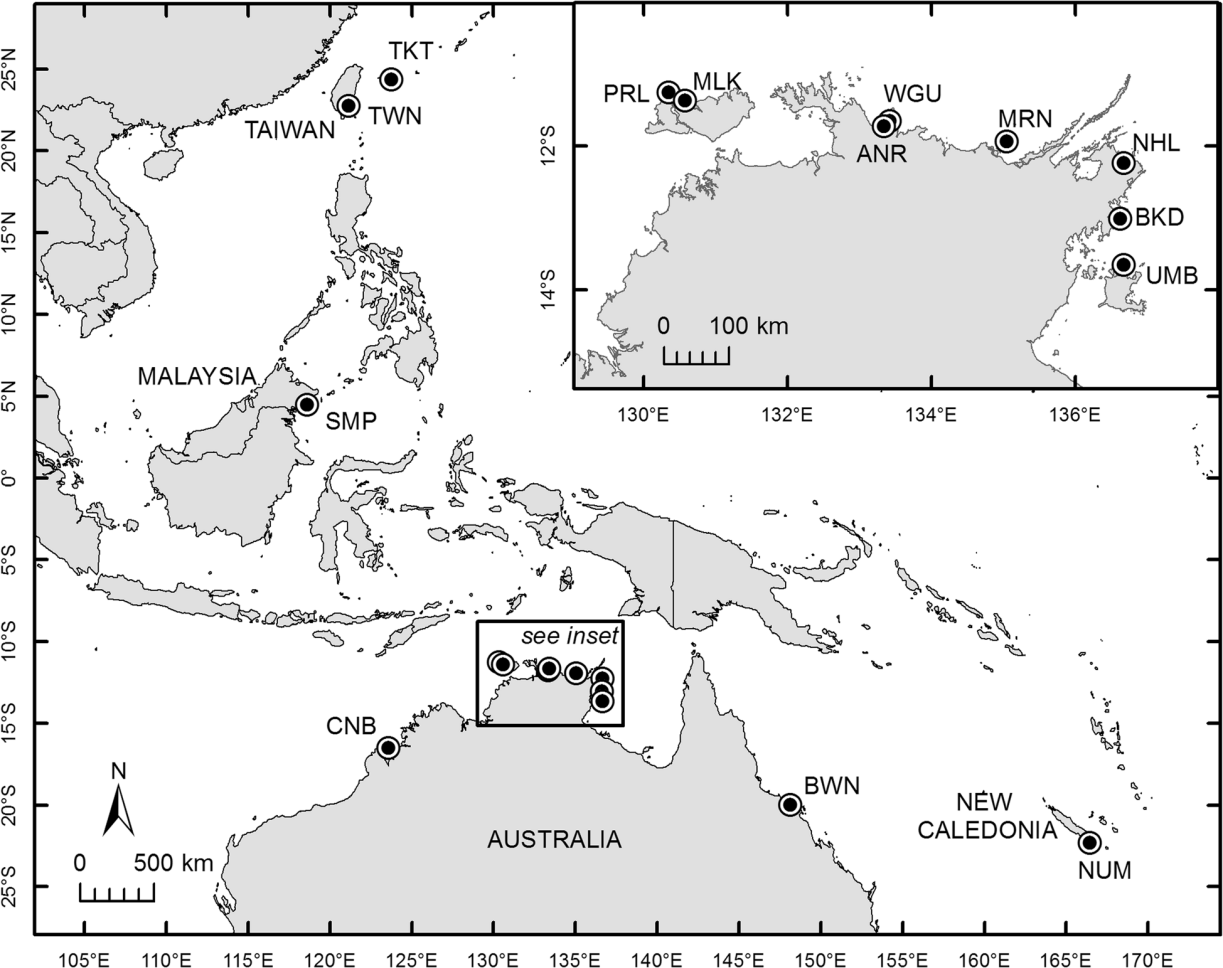
Map showing locations from which oysters (*Saccostrea echinata*) were sampled (circle markers). SMP, Semporna (4°29′6″N 118°36′12″E); TWN, Taiwan (22°45′24″N 121°09′02″E); TKT, Taketomi Island (24°21′36″N 123°44′60″E); CNB, Cone Bay (16°30′19″S 123°34′55″E); PRL, Pirlangimpi (11°15′07″S 130°20′53″E); MLK, Milikapiti (11°22′5″S 130°34′21″E); ANR, Anuru Bay (11°43′23″S 133°20′35″E); WGU, Wigu (11°38′49″S 133°25′23″E); MRN, Mooroongga Island (11°56′06″S 135°02′47″E); NHL, Nhulunbuy (12°14′14″S 136°40′30″E); BKD, Bukudal (13°00′40″S 136°37′41″E); UMB, Umbakumba (13°39′03″S 136°40′45″E); BWN, Bowen (19°56′54″S 148°08′55″E); NUM, Noumea (22°17′21″S, 166°26′36″E)

The mitochondrial cytochrome c oxidase subunit I gene (COI) data set comprised 273 sequences of 594 base pair (bp) length, which contained 74 different haplotypes. Haplotype and nucleotide diversities were high (0.887–1.000 and 0.005–0.008, respectively) and similar among all sample locations except Bowen, which were markedly lower (0.571 and 0.001, respectively) (Fig. 1; Table 1). Anuru Bay had the highest haplotype diversity (1.000 ± 0.022); every individual had a different haplotype (Table 1). Noumea had the highest number of private haplotypes (9), while Bukudal had the lowest (zero). No haplotypes were shared among all locations (Table 1). Kimura 2-parameter (K2P) distance between all localities in this study and from those obtained from GenBank (i.e. Malaysia, Taiwan, Japan and Western Australia) are provided in Additional file 1.

**Table 1:** Sample sizes and summary genetic diversity statistics for samples of *Saccostrea echinata* at a 594 base pair region of mitochondrial COI. Significance (*P* < 0.05) is indicated by bold values

	PRL	MLK	ANR	WGU	MRN	NHL	BKD	UMB	BWN	NUM	ALL
*N*_*COI*_	34	31	16	37	30	30	21	30	15	29	273
*h* (± SD)	0.954 ± 0.018	0.951 ± 0.021	1.000 ± 0.022	0.955 ± 0.016	0.966 ± 0.018	0.913 ± 0.030	0.895 ± 0.038	0.901 ± 0.034	0.571 ± 0.149	0.887 ± 0.041	0.949 ± 0.006
*N*_h_	19	18	16	19	20	13	9	15	6	14	74
*ph*	5	3	4	3	5	5	0	7	3	9	n.a.
*S*	22	25	23	26	29	18	14	20	5	14	72
*π* (± SD)	0.006 ± 0.001	0.006 ± 0.001	0.008 ± 0.001	0.007 ± 0.001	0.008 ± 0.001	0.005 ± 0.001	0.007 ± 0.001	0.005 ± 0.001	0.001 ± 0.001	0.004 ± 0.000	0.006 ± 0.000
Tajima’s *D*	−0.999	−1.656	−1.138	−1.157	−1.382	−0.998	0.419	−1.473	−1.451	− 1.090	−2.030
*P*_*TD*_	0.176	**0.033**	0.098	0.126	0.069	0.154	0.693	0.053	0.080	0.144	**0.000**
Fu’s *Fs*	−9.364	−10.084	−13.421	−7.789	−10.731	−3.831	−0.361	−6.940	−3.235	−7.186	−25.638
*P*_*Fs*_	**0.000**	**0.001**	**0.000**	**0.001**	**0.001**	**0.033**	0.450	**0.001**	**0.000**	**0.000**	**0.000**

*N*_*COI*_, sample size; *h* (± SD), haplotype diversity (± standard deviation); *N*_h_, number of haplotypes; *ph*, number of private haplotypes; *S*, number of polymorphic sites; *π* (± SD), nucleotide diversity (± standard deviation); *P*_*TD*_, probability of Tajima’s *D*; *P*_*Fs*_, Probability of Fu’s *Fs*. PRL, Pirlangimpi; MLK, Milikapiti; ANR, Anuru Bay; WGU, Wigu; MRN, Mooroongga Island; NHL, Nhulunbuy; BKD, Bukudal; UMB, Umbakumba; BWN, Bowen; NUM, Noumea

**Fig. 2:**
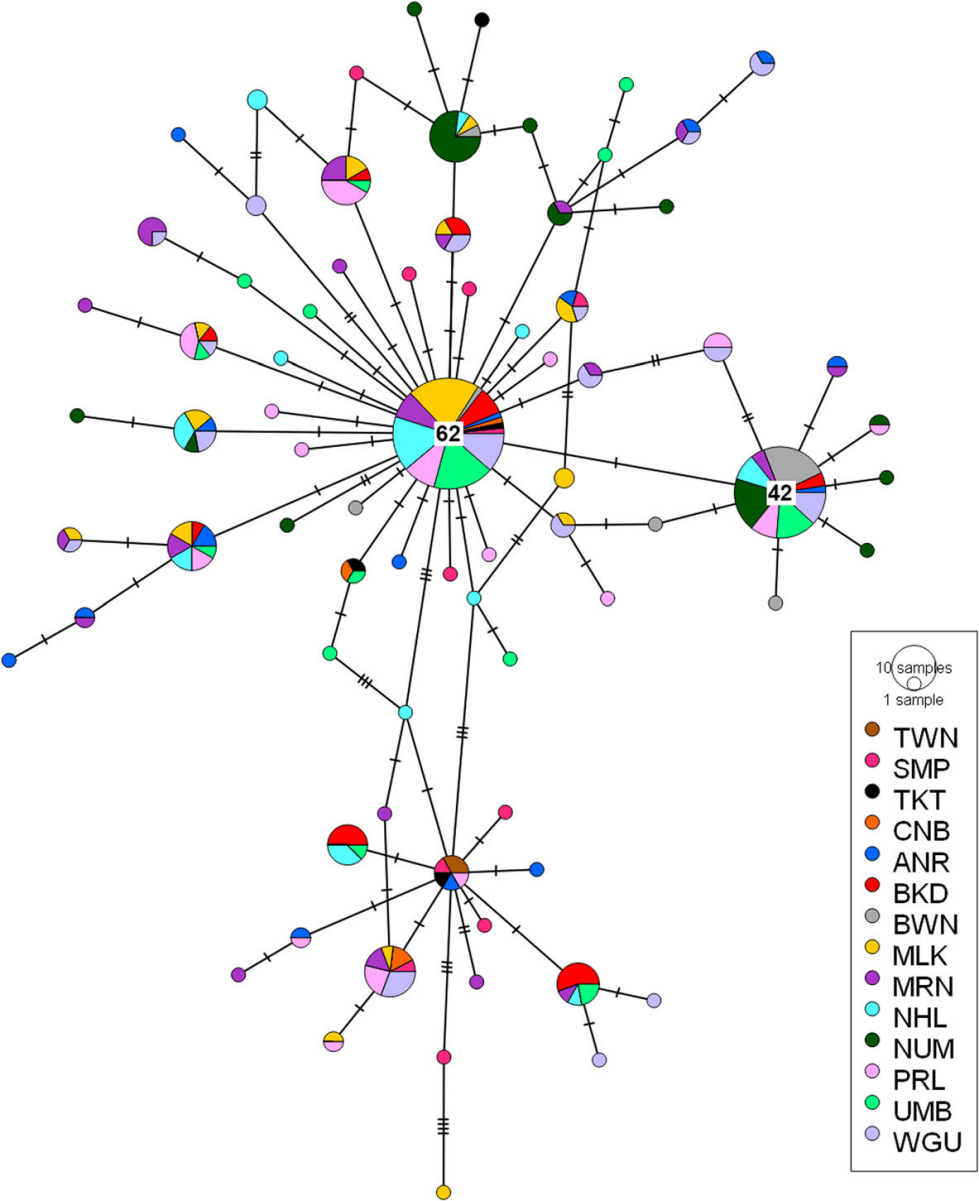
Median joining haplotype network of *Saccostrea echinata* mitochondrial COI sequence data. Each bar on the branch corresponds to a single nucleotide substitution. Larger circles contain specific frequency size

Pairwise ϕ_ST_ estimates of haplotype frequency between localities showed Bowen and Noumea (except for Noumea and Anuru Bay) as significantly different from all other populations (Table 2). Bukudal was also significantly different from both Pirlangimpi and Wigu, and Nhulunbuy was significantly different from Pirlangimpi. No other localities were different from one another. However, the level of genetic differentiation did not depend on the geographic distance separating sample locations (Mantel test, *P* = 0.104, *R*^*2*^ = 0.520). Likewise, the haplotype network indicates lack of population genetic structure between populations and does not support geographical haplotype groups (Fig. 2). The haplotype network showed two larger ‘ancestral’ haplotypes with many (28 and 7) unique haplotypes radiating from them (Fig. 2). Analyses of all locations together resulted in negative mean Tajima’s *D* and Fu’s *F*s that were significant (Table 2). Negative Tajima’s *D* indicates an excess of low frequency polymorphisms and negative Fu’s *F*s indicates an excess of alleles expected from recent directional selection or population growth [24, 31]. Phylogenetic analysis with ostreid COI sequences placed all *S. echinata* samples obtained in this study within ‘Lineage J’ as nominated by Sekino and Yamashita [32] (Additional file 2).

**Table 2:** Pairwise ϕ_ST_ estimates of haplotype frequency between each of the 10 *Saccostrea echinata* populations (below diagonal and corresponding *P*-values of ϕ_ST_ estimates (above diagonal)

	PRL	MLK	ANR	WGU	MRN	NHL	BKD	UMB	BWN	NUM
PRL	**–**	0.252	0.279	0.288	0.505	**0.000**	**0.000**	0.081	**0.000**	**0.000**
MLK	0.006	**–**	0.261	0.198	0.252	0.081	0.018	0.063	**0.000**	**0.000**
ANR	0.004	0.003	**–**	0.459	0.631	0.045	0.027	0.009	**0.000**	0.018
WGU	0.003	0.007	−0.002	**–**	0.387	0.027	**0.000**	0.027	**0.000**	**0.000**
MRN	−0.002	0.003	−0.005	0.000	**–**	0.036	0.054	0.027	**0.000**	**0.000**
NHL	**0.030**	0.013	0.021	0.027	0.026	**–**	0.234	0.784	**0.000**	**0.000**
BKD	**0.040**	0.037	0.039	**0.043**	0.027	0.011	**–**	0.297	**0.000**	**0.009**
UMB	0.019	0.020	0.029	0.025	0.021	−0.010	0.011	**–**	**0.000**	**0.000**
BWN	**0.150**	**0.211**	**0.178**	**0.139**	**0.175**	**0.154**	**0.200**	**0.121**	**–**	**0.009**
NUM	**0.048**	**0.073**	0.040	**0.041**	**0.054**	**0.056**	**0.085**	**0.054**	**0.080**	**–**

Significance (*P* < 0.05) following sequential Bonferroni correction (Rice 1989) is indicated in bold, with corresponding ϕ_ST_ values also in bold. PRL, Pirlangimpi; MLK, Milikapiti; ANR, Anuru Bay; WGU, Wigu; MRN, Mooroongga Island; NHL, Nhulunbuy; BKD, Bukudal; UMB, Umbakumba; BWN, Bowen; NUM, Noumea

### SNP data

#### Genotyping

Approximately 761 million sequence reads of 150 bp each were obtained, filtered and trimmed from the eight lanes of Illumina NextSeq500 sequencing. From these, approximately 15 million double-digest restriction site-associated DNA (ddRAD) loci from 282 individuals were identified. The data set was then filtered to 27,887 SNPs from 272 individuals which were used for further analyses (Table 3). BAYESCAN 2.1 and *PCAdapt* detected 51 and 483 outliers, respectively. Of these, 31 loci were detected by both methods and considered as candidate adaptive loci (Table 3). Subsequent analyses were conducted for the putatively neutral data set of 27,856 loci and the candidate adaptive data set of 31 loci.

**Table 3:** Number of SNPs retained after each filtering step for the black-lip rock oyster, *Saccostrea echinata*

Step	SNP count (number samples)
Stacks catalogue	15,674,503 (282 samples)
Genotyped	
> 70% of the samples	44,904 (282 samples)
> 50% of the locations	
H_OBS_ < 0.5	
Local MAF > 0.01	
Loci < 2 alleles	35,811
QC in R	
VCF conversion	35,152
Missing per SNP < 70%	27,887 (280 samples)
Missing per individual < 50%	27,887 (272 samples)
Outlier detection	
BAYESCAN outliers	51
PCAdapt outliers	483
Outliers identified with both methods	31
Putatively neutral	27,856

#### Genetic diversity and population structure

The number of alleles and the allelic richness were similar among localities; the number of alleles ranged from 32,023–45,861 (neutral) and 37–55 (candidate adaptive), and the allelic richness ranged from 1.083–1.444 (neutral) and 1.130–1.851 (candidate adaptive). Bowen had the lowest number of alleles, with 27,585 (neutral) and 28 (candidate adaptive), and an allelic richness of 0.972 (neutral) and 0.892 (candidate adaptive) (Table 4). Observed heterozygosity was lower than expected heterozygosity for all populations (Table 4). Mean expected and observed heterozygosity were higher for the candidate adaptive SNPs than the neutral SNPs and the inbreeding coefficient was similar and positive among localities (Table 4). Estimates of effective population size ranged from 212.8–infinity (neutral) and varied among sampling locations (Table 4). Infinite effective population size estimates and confidence intervals may be due to larger than expected sampling error [33]. Levels of population differentiation across localities were high with 39 out of 45 pairwise comparisons significantly different than zero (Table 5). Pairwise comparisons for the neutral data set ranged from zero (Milikapiti vs Pirlangimpi, Umbakumba vs. Nhulunbuy, and Bukudal vs. Nhulunbuy) to 0.058 (Noumea vs. Anuru Bay and Noumea vs. Wigu). Pairwise estimates that were not significant occurred between some of the Northern Territory populations, indicating greater homogeneity between these populations.

**Table 4:** Sample sizes and summary genetic diversity statistics for samples of *Saccostrea echinata* from 10 localities based on 27,896 putatively neutral and 31 candidate adaptive SNPs

		PRL	MLK	ANR	WGU	MRN	NHL	BKD	UMB	BWN	NUM
*N*_*SNP*_		37	24	19	37	30	30	16	28	15	26
Source		Wild	Wild	Wild	Wild	Wild	Wild	Wild	Wild	Farm (wild origin)	Wild
*N*_*E*_ *[95% C.I.]*	Neutral	6164.2 [4536.1–9613.2]	25,655.2 [7621.2–∞]	∞ [∞–∞]	2751.7 [2335.-53,243.4]	212.8 [209.–5216.3]	2466.4 [2099.7–2987.7]	∞ [∞–∞]	3566.9 [2560.8–5869.9]	∞ [∞–∞]	2521.5 [2068.6–3227.0]
*H*_*E*_	Neutral	0.107	0.105	0.109	0.105	0.107	0.105	0.101	0.097	0.088	0.102
	Adaptive	0.273	0.218	0.268	0.265	0.306	0.316	0.325	0.281	0.103	0.128
*H*_*0*_	Neutral	0.070	0.070	0.076	0.068	0.071	0.071	0.070	0.064	0.060	0.070
	Adaptive	0.185	0.174	0.175	0.175	0.181	0.222	0.184	0.230	0.054	0.095
*F*_*IS*_	Neutral	0.341	0.336	0.307	0.349	0.335	0.322	0.308	0.335	0.316	0.312
	Adaptive	0.321	0.205	0.348	0.342	0.407	0.298	0.435	0.182	0.479	0.258
*A*	Neutral	45,285	41,821	42,084	45,861	44,313	42,674	32,023	33,115	27,585	38,633
	Adaptive	54	48	51	55	59	50	42	43	28	37
*A*_*R*_	Neutral	1.422	1.382	1.430	1.444	1.426	1.388	1.112	1.083	0.972	1.299
	Adaptive	1.642	1.484	1.612	1.689	1.851	1.558	1.315	1.315	0.892	1.130

*N*_*SNP,*_ sample size; *N*_*E*_ *[95% C.I.],* effective population size [95% confidence interval]; *H*_*E*_*,* expected heterozygosity; *H*_*O*_*,* observed heterozygosity; *F*_*IS*_*,* inbreeding coefficient (fixation index); *A*, number of alleles; *A*_*R*_, allelic richness. *PRL*, Pirlangimpi; *MLK*, Milikapiti; *ANR*, Anuru Bay; *WGU*, Wigu; *MRN*, Mooroongga Island; *NHL*, Nhulunbuy; *BKD*, Bukudal; *UMB*, Umbakumba; *BWN*, Bowen; *NUM*, Noumea

**Table 5:** Pairwise *F*_ST_ estimates between each of the 10 *Saccostrea echinata* populations based on 27,896 putatively neutral SNPs (below diagonal and corresponding *P*-values of *F*_ST_ estimates (above diagonal)

	PRL	MLK	ANR	WGU	MRN	NHL	BKD	UMB	BWN	NUM
PRL	**–**	0.240	**0.003**	**0.001**	**0.001**	**0.001**	**0.001**	**0.001**	**0.001**	**0.001**
MLK	0.000	**–**	**0.001**	**0.001**	**0.002**	**0.001**	**0.001**	**0.001**	**0.001**	**0.001**
ANR	**0.002**	**0.003**	**–**	0.141	0.050	**0.001**	**0.001**	**0.001**	**0.001**	**0.001**
WGU	0.002	**0.002**	0.001	**–**	**0.001**	**0.001**	**0.001**	**0.001**	**0.001**	**0.001**
MRN	**0.003**	**0.003**	0.002	**0.002**	**–**	**0.001**	**0.001**	**0.001**	**0.001**	**0.001**
NHL	**0.031**	**0.031**	**0.032**	**0.032**	**0.029**	**–**	0.668	0.285	**0.001**	**0.001**
BKD	**0.027**	**0.028**	**0.029**	**0.027**	**0.024**	0.000	**–**	0.247	**0.001**	**0.001**
UMB	**0.030**	**0.030**	**0.032**	**0.031**	**0.027**	0.000	0.001	**–**	**0.001**	**0.001**
BWN	**0.052**	**0.053**	**0.055**	**0.052**	**0.049**	**0.052**	**0.049**	**0.049**	**–**	**0.001**
NUM	**0.056**	**0.056**	**0.058**	**0.058**	**0.056**	**0.056**	**0.053**	**0.054**	**0.041**	**–**

Significance (*P* < 0.05) following sequential Bonferroni correction (Rice 1989) is indicated in bold, with corresponding *F*_ST_ values also in bold. *PRL*, Pirlangimpi; *MLK*, Milikapiti; *ANR*, Anuru Bay; *WGU*, Wigu; *MRN*, Mooroongga Island; *NHL*, Nhulunbuy; *BKD*, Bukudal; *UMB*, Umbakumba; *BWN*, Bowen; *NUM*, Noumea

**Fig. 3:**
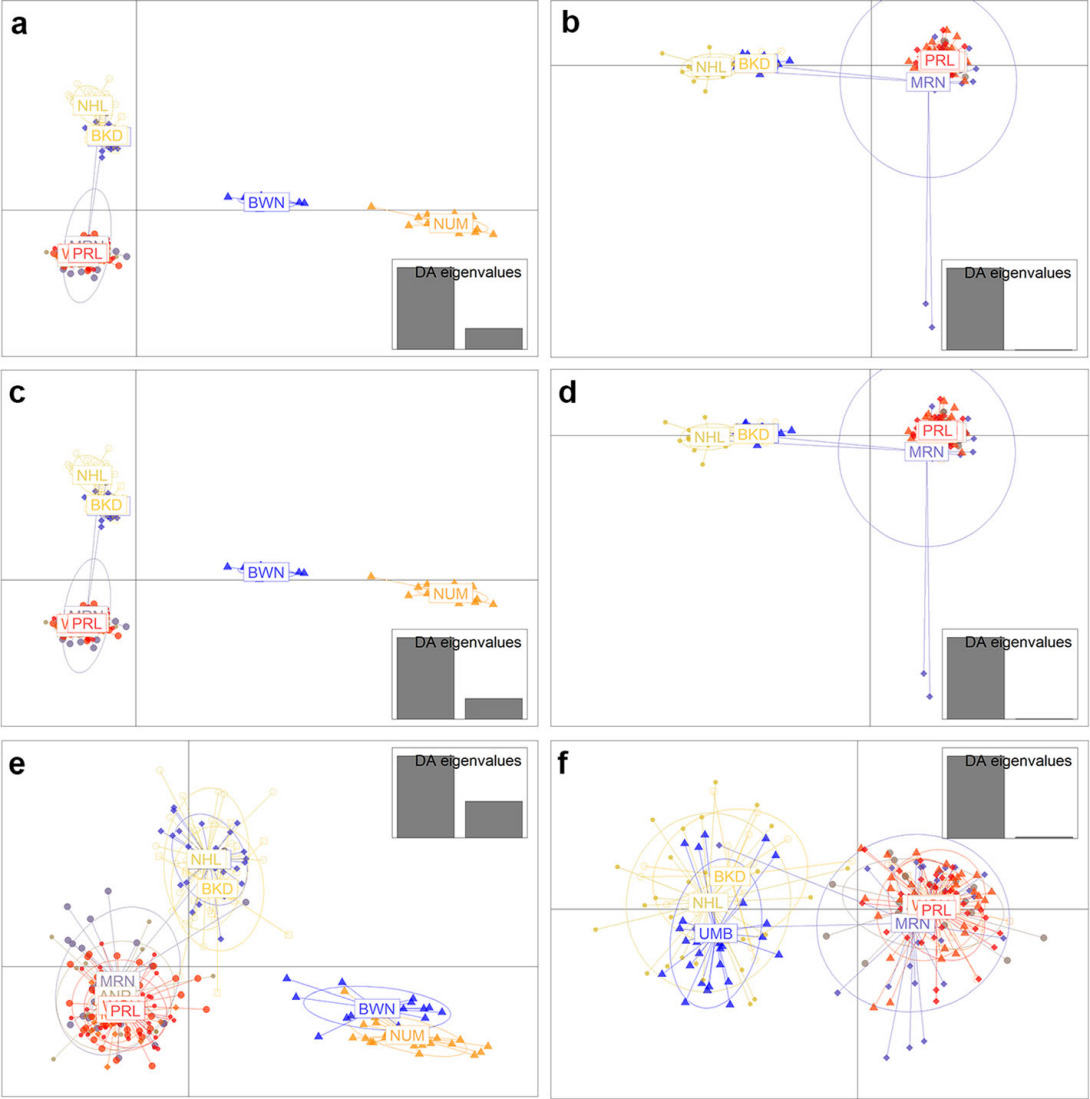
Discriminant analyses of principal components (DAPC) of *Saccostrea echinata* samples by sampling locality. (**a**) For all 27,887 SNPs and all sample locations (**b**) For all 27,887 SNPs and only Northern Territory sample locations (**c**) For all 27,856 putatively neutral SNPs and all sample locations (**d**) For all 27,856 putatively neutral SNPs and only Northern Territory sample locations (**e**) For all 31 candidate adaptive SNPs and all sample locations (**f**) For all 31 candidate adaptive SNPs and only Northern Territory sample locations. PRL, Pirlangimpi; MLK, Milikapiti; ANR, Anuru Bay; WGU, Wigu; MRN, Mooroongga Island; NHL, Nhulunbuy; BKD, Bukudal; UMB, Umbakumba; BWN, Bowen; NUM, Noumea

While both neutral and candidate adaptive SNPs detected population structure between sampling locations, the neutral loci detected greater differentiation (Fig. 3a–f). Discriminant analysis of principal components (DAPC) of all 10 sampling locations using all SNPs detected four clusters, with Noumea and Bowen forming their own clusters and the remaining localities from the Northern Territory, Australia separating into two clusters (Fig 3a). Analyses of only the eight Northern Territory sampling locations showed some mixing between the two clusters by the Mooroongga population (Fig. 3b). This pattern remained when analysing neutral and candidate adaptive SNPs separately (Fig. 3d,f). There was a significant positive correlation between genetic differentiation and the geographic distance separating sample locations (Mantel test, *P* < 0.001, *R*^*2*^ = 0.798).

Individuals were assigned to their region of origin with very high success; 98.6 and 100% of individuals were correctly assigned to population of origin, with both 10,000 SNP data sets. Two migrant individuals were detected and both were from the west population, specifically the Mooroongga sample location.

#### Functional annotation

The alignment of the 31 candidate sequences to the complete black-lip rock oyster transcriptome resulted in 19 hits. Three of these were successfully identified as belonging to known genes and are presented in Table 6: (1) SNP *2350* is situated in the gene Kmt5a, which is required for cell proliferation, probably by contributing to the maintenance of higher-order structure of DNA during mitosis; (2) SNP *18035* is situated in the gene FAXC that may play a role in neural, axonal development; and (3) SNP *34199* which is situated in the sex peptide receptor (SPR) gene. The SPR gene had the most well-described gene ontology annotation terms and has been reported to be involved in controlling reproductive and sleep behaviours in fruit flies (*Drosophila melanogaster*) [34, 35].

**Table 6:** Characterisation of high-quality BLAST matches obtained in comparison with *Saccostrea echinata* 31 candidate adaptive SNPs against *Saccostrea* lineage J transcriptome (McDougall 2018)

SNP #	UniProt ID	SNP ID	Protein names	Gene names	Transcript ID	Species	E-value	Hit Length	Uniprot GO
02	KMT5A_MOUSE	2350	N-lysine methyltransferase KMT5A	Kmt5a	BL_TRINITY_DN42314_c0_g1_i4	*Mus musculus (Mouse)*	6 × 10^−56^	138	**Molecular function** • histone-lysine N-methyltransferase activity • p53 binding • protein-lysine N-methyltransferase activity • transcription corepressor activity **Biological process** • cell cycle • cell division • negative regulation of transcription, DNA-templated • negative regulation of transcription by RNA polymerase II • peptidyl-lysine monomethylation • regulation of DNA damage response, signal transduction by p53 class mediator
07	FAXC_HUMAN	18,035	Failed axon connections homolog	FAXC	BL_TRINITY_DN51192_c2_g1_i5	*Homo sapiens (Human)*	3 × 10^−22^	68	• May play a role in axonal development
09	SPR_DROME	34,199	Sex peptide receptor	SPR	BL_TRINITY_DN29536_c0_g1_i1	*Drosophila melanogaster (Fruit fly)*	8 × 10^−67^	140	**Molecular function** • G protein-coupled peptide receptor activity **Biological process** • G protein-coupled receptor signalling pathway • negative regulation of female receptivity, post-mating • regulation of post-mating oviposition

## Discussion

This is the first study to investigate the population genetics of *S. echinata* and it utilises both mitochondrial and nuclear DNA markers. Results support the existence of genetically distinct populations of *S. echinata*, and this has implications for the management of wild and farmed stocks of this new aquaculture species, that should be considered as multiple management units.

Results from the mitochondrial COI analyses, are consistent with those of McDougall [36], and the observational data of Thomson [26], in that they support a broad tropical Indo-Pacific distribution of *S. echinata*; from at least Taketomi Island in Japan, to Noumea in New Caledonia, and across northern Australia; from Cone Bay in Western Australia, to Bowen in Queensland. Few studies have employed genetic data to assist in investigating the distribution of tropical oyster species within the Indo-Pacific region. The tropical rock oyster, *C. iredalei*, has been confirmed to occur throughout Malaysia, based on mitochondrial COI analyses [24]. Similar research has also been conducted with the tropical black-lip pearl oyster, *Pinctada margaritifera,* which confirmed broad Indo-Pacific distribution, based on SNP analyses [14]. Genetic confirmation of the geographic range of *S. echinata* is important for countries interested in aquaculture of this species, particularly those interested in hatchery production of spat, because culture conditions and techniques are species-specific [37, 38]. A recent review of the diversity and evolution of oysters (both temperate and tropical species) with a focus on molecular studies by Guo et al. [39] highlighted that balancing selection is a major force in shaping genetic variation among oyster populations. Strong balancing selection results from; different life stages (planktonic larvae and sessile adults), long-distance dispersal potential, and fluctuating environmental conditions, and results in high levels of genetic diversity [40, 41]. High genetic diversity has been shown across oyster populations that are panmictic, such as *Crassostrea gigas* in Japan [42], and highly divergent, such as *C. virginica* in America [43]. In the current study, analyses of mitochondrial COI data showed high haplotype and nucleotide diversities (excluding the Bowen location), that are similar to those reported for other molluscs, such as the oyster *C. iredalei* [24], the clam *Anomalocardia brasiliana* [44], and the Hawaiian limpet *Cellana talcosa* [45]. These studies [44, 45] have been compared in a summary of published haplotype and nucleotide diversity estimates by Goodall-Copestake et al. [46], to provide a standardised reference for comparison and improve the consistency of reporting for diversity estimates. High diversity estimates in wild populations, as reported in the current study, are a positive attribute for hatchery-based aquaculture and support the development of selective breeding programs [6, 10].

Relatively low haplotype and nucleotide diversity indices detected in Bowen samples may be due to unintentional sampling bias. These individuals were collected from an oyster farm and it is possible that they may have been collected from a single or few cohort(s). It is also probable that the Bowen population is close to the edge of the species range and many taxa show a general decline in genetic diversity towards range margins; a pattern known as the core-periphery hypotheses [14, 47, 48]. Despite Bowen and Noumea being the most distant sample locations, mitochondrial COI analyses did not support isolation by distance, and the haplotype network for *S. echinata* in this study did not support geographical haplotype groups. The ‘star-like’ haplotype network and congruence between Tajima’s *D* and Fu’s *F*s overall negative and significant *P*-values suggest a recent population expansion after a bottleneck [24, 49].

In contrast to the lack of structure between sampled populations of *S. echinata* shown by mitochondrial COI analyses, the SNP analyses showed high levels of population subdivision. Greater differentiation of nuclear SNP markers, compared with mitochondrial COI markers, was similarly reported by Varney et al. [50] in a population genetic investigation of the eastern oyster, *C. virginica*, in the Gulf of Mexico. Mitochondrial DNA analysis is a useful first-pass marker for investigating genetic structure, however, it is not a suitable marker for the detection of fine spatial genetic structure [51], and it is therefore important to follow-up with higher resolution genomic markers [52], such as genome-wide SNPs [53–55].

In the current study, the SNP analyses clearly showed that the *S. echinata* populations sampled do not belong to a single panmictic unit. At a broad scale, *S. echinata* populations in Bowen and Noumea displayed substantial and significant divergence from all other populations. Within the Northern Territory, Australia, low but significant differentiation was observed among some of the sample locations and DAPC analyses identified two genetic clusters in this region (here termed ‘west’ and ‘east’). Some mixing was detected, by the DAPC analyses, between the west and east clusters in the Northern Territory, which was further confirmed by the detection of two migrant individuals at Mooroongga, which is part of the west cluster, that were genetically most similar to the Nhulunbuy sample location, which is part of the east cluster. Both the structure shown in the DAPC analyses and a significant positive correlation between genetic and geographic distance support isolation by distance occurring in sampled *S. echinata* populations.

**Fig. 4:**
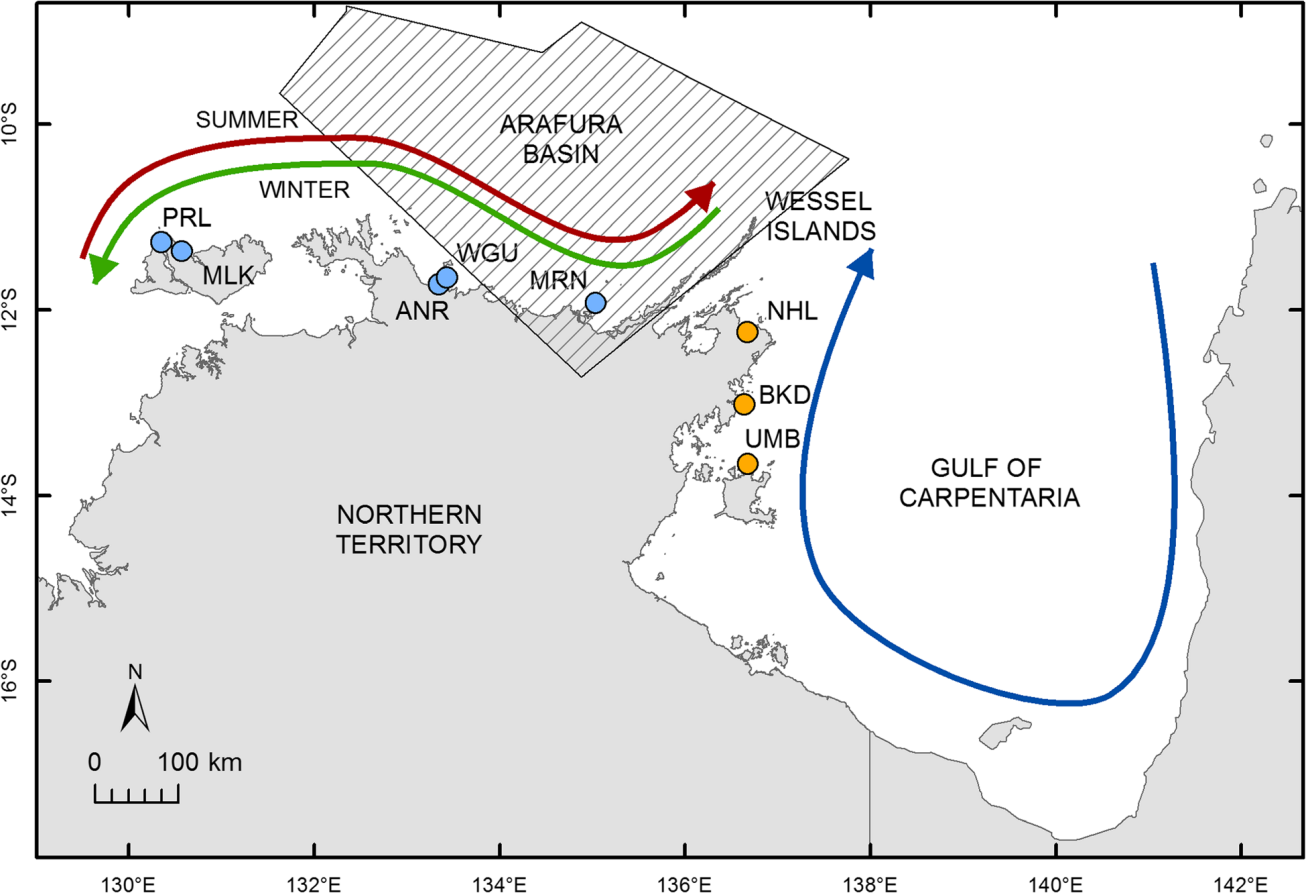
Map showing locations from which oysters (*Saccostrea echinata*) were sampled within the Northern Territory, Australia. Different coloured circle markers indicate two genetic clusters (1) the west cluster (blue circle markers); PRL, Pirlangimpi (11°15′07″S 130°20′53″E); MLK, Milikapiti (11°22′5″S 130°34′21″E); ANR, Anuru Bay (11°43′23″S 133°20′35″E); WGU, Wigu (11°38′49″S 133°25′23″E); and MRN, Mooroongga Island (11°56′06″S 135°02′47″E); and (2) the east cluster (orange circle markers); NHL, Nhulunbuy (12°14′14″S 136°40′30″E); BKD, Bukudal (13°00′40″S 136°37′41″E); and UMB, Umbakumba (13°39′03″S 136°40′45″E). The dominant near-surface current circulation patterns and location of the Arafura Basin (indicated by the lined polygon) are illustrated (adapted from Schiller [56] and Geoscience Australia [57])

Distinct genetic clusters within the Northern Territory suggest that larval dispersal is limited to within these two regions. These two clusters occur either side of the Wessel Islands, a chain of small islands extending 120 km northeast from the Napier Peninsula (Fig. 4), that are a potential physical barrier to *S. echinata* larval dispersal and thus gene flow. Furthermore, this island chain would have been a land barrier around seven thousand years ago, when sea levels were lower, which may be reflected in the genetic clusters identified [58]. In a similar study investigating population genetics of the sea cucumber, *Holothuria scabra,* in northern Australia, Gardner et al. [59] reported the same genetic clusters either side of the Wessel Islands. They suggested a hydrological barrier exists, due to prevailing surface currents (Fig. 4); currents on the west side carry larvae westward and on the ‘east’ side a circular current in the Gulf may entrain larvae, restricting the passive dispersal of *H. scabra* larvae, which have a similar larval duration to larvae of *S. echinata* [29, 59]. Gardner et al. [59] also proposed that the Arafura Basin, situated west of the Wessel Islands (Fig. 4), may act as a benthic barrier to larval dispersal, because rivers that drain into the Arafura Basin create an environment of low salinity [60]. The congruence between the findings of Gardner et al. [59] and the current study, support the need for further investigation to determine mechanisms underlying shared genetic patterns; such investigations may include a multi-species phylogeographic study of the region [52] and more sampling of *S. echinata* throughout the Gulf of Carpentaria.

When undertaking population genetic analyses with SNPs it is important to consider both neutral markers, which provide useful information about neutral evolutionary processes, and candidate adaptive markers which are more likely to display signatures of selection [54]. In the current study neutral and candidate adaptive SNPs were analysed separately and together (when appropriate), and no major conflicts between resulting data sets were detected. One advantage for determining candidate adaptive SNPs is that they can identify genes under selection, and if these genes are known, then gene ontology may reveal locally important adaptations [53, 55]. In the current study of *S. echinata*, three candidate adaptive SNPs were successfully identified as belonging to known genes, and gene ontology was well described for the SPR gene. The SPR gene has been studied in fruit flies (*D. melanogaster*) and has been shown to be important for determining reproductive behaviour [34, 61, 62] and sleep stabilisation [35]. A molecular function of this gene is G protein-coupled peptide receptor activity which can be important in immunity, protein activation, and other molecular functions [34]. The SPR gene may play a role in adaptive differences between the *S. echinata* populations studied, such as differences in reproductive behaviours (e.g. the timing of reproductive cycles or of spawning). Further research is required to better understand the role of the SPR gene in relation to seascape genomics of *S. echinata.*

### Management implications

Genetic information is an important part of fisheries and aquaculture management policy and has been used to identify management units for many commercially important species, including green-lip abalone (*Haliotis laevigata*) [2, 63], mangrove oysters (*Crassostrea* spp.) [64], Atlantic cod (*Gadus morhua*) [53], and black-lip pearl oysters (*Pinctada margaritifera*) [14]. Given this study’s findings of significant population differentiation and the presence of signatures of selection, the data supports the existence of genetically distinct populations of *S. echinata*. This suggests that management of wild and farmed *S. echinata* stocks should be based upon multiple management units, and new areas interested in farming this species should undergo genetic stock assessments to determine optimal management practises. Within the Northern Territory, Australia where most of the sample locations in this study occur, and where *S. echinata* aquaculture development is focused, two genetically distinct clusters exist, which may be differentially adapted to local environmental conditions. It is recommended that these distinct clusters be considered as management units; when broodstock are being selected for hatchery production, for the translocation of wild or hatchery-produced spat, and for any translocations of farmed stocks.

## Conclusions

Use of both mitochondrial and nuclear DNA markers to assess the population genetics of *S. echinata* across northern Australia has made data on population genetic structure and connectivity available. This study confirmed that despite the potential for high levels of gene flow, *S. echinata* do not belong to a single panmictic unit. The development of management policy for culture practices and translocation of farmed oysters will become increasingly important as hatchery production develops and aquaculture operations expand. Furthermore, this study has provided high quality genome-wide SNP marker sets that can be utilised for future genetic research, such as the use of genetics in stock improvements (e.g. selective breeding programs), and to improve understanding of locally important adaptations in wild oyster populations.

## Methods

### Sample collection and genomic DNA extraction

In total, 273 oysters were sampled across 10 sampling sites; nine in Australia (from the Northern Territory and Queensland) and one in New Caledonia (Fig. 1). The sites chosen within Australia were those under consideration for commercial oyster farming. The New Caledonian sample collection was opportunistic, however, the site is also under consideration for farming. Between 15 and 37 oysters were collected randomly at each site, during low tide, from intertidal wild stocks before being transported live in a damp hessian bag, inside a foam box to the Northern Territory Government, Department of Primary Industry and Resources, Darwin Aquaculture Centre. A 0.5 cm^2^ of adductor muscle was excised from live oysters and stored in 96% ethanol in an Eppendorf tube. Exceptions to this method include samples collected from Bowen, Australia (BWN, Fig. 1) where oysters were collected from farmed stocks, and samples collected from Noumea, New Caledonia (NUM, Fig. 1) where processing of samples occurred in country. Genomic DNA (gDNA) was extracted using the DNeasy Blood and Tissue kit (QIAGEN®, Germany) following the manufacturer’s instructions with a final elution of 100 μL.

### Analyses of mitochondrial COI data

#### Molecular techniques

A 594 bp fragment of the mitochondrial COI gene was amplified using the polymerase chain reaction (PCR) protocol. The ‘Folmer’ primers [9] were used and reaction conditions were: 1 cycle of 94 °C for 1 min, followed by 5 cycles of 94 °C for 40 s, 45 °C for 40 s, 72 °C for 1 min, followed by 35 cycles of 94 °C for 40 s, 51 °C for 40 s, 72 °C for 1 min and concluding with a final extension of 72 °C for 5 min. Integrity of gDNA was determined through gel electrophoresis.

#### Data analyses

Sequences were aligned using Geneious Alignment implemented within Geneious 11.1.5 (http://www.geneious.com/) and trimmed (sequence accession numbers provided in Additional file 3). Sequences were grouped into source localities for data analyses according to their geographical proximity (Fig. 1). Haplotype (*h*) and nucleotide (*π*) diversities were calculated using DnaSP 6.12.01 [65]. Tajima’s *D* and Fu’s *Fs* neutrality tests were performed in the program ARLEQUIN 3.5.2.2 [31, 66, 67]. Pairwise ϕ_ST_ estimates of haplotype frequency between localities were also calculated using ARLEQUIN 3.5.2.2. To determine whether an isolation by distance pattern was prevalent in *S. echinata*, a genetic distance (ϕ_ST_) matrix was compared with a geographic distance (km) matrix using the R package *ade4* [68]. Geographical distances were estimated as the shortest distance by sea between the locality pairs as determined by Google Earth. A median joining haplotype network was generated from the mitochondrial COI sequence data using PopART [69, 70] with the default settings. Sequences of *S. echinata* from previous studies (*n* = 21) [32, 71] were obtained from GenBank and also included in the network analyses. Sampling sites of these sequences include Semporna in Malaysia, Taiwan, and Taketomi Island in Japan (coordinates listed in Fig. 1). Sequences of *S. echinata* were also obtained from the Government of Western Australia (*n* = 4) from the sampling site Cone Bay (coordinates listed in Fig. 1). Evolutionary distances were estimated using the program MEGA-X with the K2P model [72]. Mitochondrial COI sequences were aligned with other partial ostreid COI sequences obtained from McDougall [35] and phylogenetic analysis was performed in Geneious 11.1.5 using the Tamura-Nei genetic distance model with the Neighbour-Joining tree building method and 100 nonparametric bootstrap replicates. Phylogenetic trees were visualised using FigTree [73].

### Analyses of SNP data

#### Molecular techniques

Double-digest restriction site-associated DNA (ddRAD) libraries were constructed following a protocol modified from Peterson et al. [74]. Briefly, 200 ng of gDNA was digested using the restriction enzymes EcoRI and NlaIII. One of 48 unique bp barcodes was ligated to each individual library. Size selection of pooled digested-ligated fragments was conducted using Blue Pippin (Sage Science, USA). Libraries were amplified using PCR and single-read, 150 bp target length sequencing was conducted on an Illumina NextSeq500 platform with 150 cycles in high-output mode at the Australia Genome Research Facility (Melbourne, Australia).

#### Bioinformatics and genotyping

Libraries were demultiplexed using the *process_radtags* program in STACKS 1.47 [75]. Reads were allowed a maximum of two nucleotide mismatches (*n* = 2) and a minimum stack depth of three (*m* = 3) (*ustacks* module in STACKS, with default parameters). Then stacks were aligned de novo with each other to create a catalogue of putative RAD tags (*cstacks* module in STACKS, with a maximum of one nucleotide mismatch allowed (*n* = 1)). In the *population* module of STACKS, and following consecutive filtering steps, SNPs were retained in at least 70% of the individuals and 50% of the locations, had a minor allele frequency of at least 10%, and heterozygosity < 0.5. Analyses were restricted to one random SNP per locus (using the *--write_random_snp* option in the populations module). SNPs with more than 70% of missing data and individuals with more than 50% missing data were also eliminated using VCFtools [76]. Details of the number of SNPs kept after each filtering step are provided in Table 3. The resulting filtered VCF file (Additional file 4) was converted into the file formats necessary for the following analyses using PGDSpider 2.1.1.5 [77].

#### Data analyses

To identify SNPs potentially under neutral and adaptive selection a combination of two outlier detection approaches were used; BAYESCAN 2.1 and *PCAdapt* R package [78]. Outlier loci detected by both BAYESCAN and *PCAdapt* were conservatively selected as candidate adaptive loci (Additional file 5) and subsequent analyses were run for the neutral and outlier datasets separately. The level of observed heterozygosity (*H*_*O*_) and expected heterozygosity (*H*_*E*_) in each locality, as well as *F* statistics were calculated using GenoDive 2.0 [79]. The number of alleles (*A*) and allelic richness (*A*_*R*_) were calculated with the R packages *PopGenReport* and *hierfstat*, respectively [80]. Effective population size (*N*_*E*_) and 95% confidence intervals were estimated using the software NeEstimator v.2.1 using the linkage disequilibrium method [33] for neutral SNPs only as the method assumes neutrality. Population structure was investigated through DAPC analyses. DAPC was performed, and results were plotted using the R package *adegenet* [81]. To determine whether an isolation by distance pattern is prevalent in *S. echinata*, a genetic distance (*F*_*ST*_) matrix was compared with a geographic distance (km) matrix using the R package *ade4* [68]. Geographical distances were estimated as the shortest distance by sea between the locality pairs as determined by Google Earth.

Assignment tests were performed in GenoDive 2.0 [79] on two data sets of 10,000 SNPs that were randomly generated from the neutral SNP data set. Individuals from Bowen and Noumea were removed and the remaining individuals from the sample locations in northern Australia were merged to form two ‘populations’ (determined by the DAPC clusters). The west population included: Pirlangimpi, Milikapiti, Anuru Bay, Wigu, and Mooroongga Island. The east population included: Nhulunbuy, Bukudal, and Umbakumba. This was done to increase the accuracy of the assignment test, as they are moderately accurate (~ 78%) for populations with low levels of genetic subdivision (*F*_*ST*_ ~ 0.04) [82]. Pairwise *F*_*ST*_ values between the two population clusters was 0.03 for both data sets of 10,000 randomly chosen SNPs. In a given genotype, when the observed frequency of any allele was zero (a missing allele), the frequency of this allele was replaced by a fixed value of 0.005 as recommended by Paetkau et al. [83], to avoid the calculation of a multilocus likelihood of zero. A null distribution of likelihood values was generated using a Monte Carlo chain [84] for 5000 permutations. The Cornuet et al. [84] algorithm, with a statistical threshold calculated separately for every population based on an alpha-value of 0.05, was used [82]. Individuals with likelihood values originating from their sampling location (*L*_*H*_) inferior to this threshold are thus defined as putative migrants.

#### Functional annotation

The basic local alignment search tool (BLAST) function, incorporated in Geneious 11.1.5, was used to BLAST the 31 candidate adaptive SNPs against the complete, annotated transcriptome of the black-lip rock oyster, referred to as *Saccostrea* lineage J by McDougall [36]. A minimal E-value threshold of 1 × 10^− 6^ and the highest ‘Grade’ (a weighted score for the hit comprised of the E-value, the pairwise identity, and the coverage) for each hit were applied to the analyses. This yielded three candidate SNPs successfully identified as belonging to known genes, contained within the UniProtKB/ Swiss-Prot database [85]. Gene ontology annotation terms were then associated with the candidate SNPs.

## Additional files

Additional file 1: This file contains a table of Kimura 2-parameter genetic distances in mitochondrial COI sequence data. (DOCX 13 kb)

Additional file 2: Phylogenetic analysis of *Saccostrea* COI sequences. Bootstrap values are given on branches, and the scale bar indicates the number of substitutions per site. The clades containing *Striostrea*, *Magallana* and *Crassostrea* COI sequences are used as outgroups. Lineages have been designated (where possible) following Lam and Morton [86] and Sekino and Yamashita [32]. (PDF 386 kb)

Additional file 3: This file contain mitochondrial COI sequence data accession numbers for 273 individuals of *S. echinata*. (XLSX 13 kb)

Additional file 4: This VCF file contains genotypic data of 272 individuals of *S. echinata* at 27,887 genome-wide SNPs. (ZIP 12536 kb)

Additional file 5: This file includes a list of identifying numbers and sequences for the 31 candidate adaptive SNPs. (DOCX 14 kb)

## Data Availability

All data generated or analysed during this study are included in this published article and its Additional files.

## Abbreviations

BLAST: basic local alignment search tool
Bp: Base pair
DAPC: Discriminant analysis of principal components
ddRAD: Double-digest restriction site-associated DNA
DNA: Deoxyribonucleic acid
gDNA: Genomic deoxyribonucleic acid
GO: Gene ontology
mitochondrial COI: Mitochondrial cytochrome c oxidase subunit I gene
PCR: Polymerase chain reaction
RADseq: Restriction-site associated DNA
SNP: Single nucleotide polymorphism
SPR gene: Sex peptide receptor gene
