# Supplementary material for: Mitochondrial and nuclear genetic analyses of the tropical black-lip rock oyster (Saccostrea echinata) reveals population subdivision and informs sustainable aquaculture development
Source: BMC Genomics. 2019 Sep 12;20:711. doi: 10.1186/s12864-019-6052-z (PMC6740020; doi:10.1186/s12864-019-6052-z)
Supplement: Supplementary file 1 — This file contains a table of Kimura 2-parameter genetic distances in mitochondrial COI sequence data. (DOCX 13 kb) [file 12864_2019_6052_MOESM1_ESM.docx]

**Additional file 1** Kimura 2-parameter genetic distances in mitochondrial COI sequence data.

|  | TWN | SMP | TKT | CNB | ANR | BKD | BWN | MLK | MRN | NHL | NUM | PRL | UMB | WGU |
| --- | --- | --- | --- | --- | --- | --- | --- | --- | --- | --- | --- | --- | --- | --- |
|  |  |  |  |  |  |  |  |  |  |  |  |  |  |  |
| TWN | 0.000 |  |  |  |  |  |  |  |  |  |  |  |  |  |
| SMP | 0.007 | 0.008 |  |  |  |  |  |  |  |  |  |  |  |  |
| TKT | 0.007 | 0.007 | 0.008 |  |  |  |  |  |  |  |  |  |  |  |
| CNB | 0.006 | 0.009 | 0.006 | 0.009 |  |  |  |  |  |  |  |  |  |  |
| ANR | 0.009 | 0.009 | 0.007 | 0.009 | 0.009 |  |  |  |  |  |  |  |  |  |
| BKD | 0.007 | 0.008 | 0.006 | 0.008 | 0.008 | 0.008 |  |  |  |  |  |  |  |  |
| BWN | 0.010 | 0.008 | 0.005 | 0.008 | 0.006 | 0.007 | 0.002 |  |  |  |  |  |  |  |
| MLK | 0.009 | 0.008 | 0.005 | 0.008 | 0.007 | 0.007 | 0.005 | 0.005 |  |  |  |  |  |  |
| MRN | 0.009 | 0.009 | 0.006 | 0.009 | 0.008 | 0.008 | 0.006 | 0.007 | 0.008 |  |  |  |  |  |
| NHL | 0.009 | 0.008 | 0.005 | 0.008 | 0.007 | 0.007 | 0.004 | 0.006 | 0.007 | 0.006 |  |  |  |  |
| NUM | 0.011 | 0.008 | 0.005 | 0.009 | 0.007 | 0.008 | 0.003 | 0.006 | 0.007 | 0.005 | 0.004 |  |  |  |
| PRL | 0.009 | 0.008 | 0.006 | 0.008 | 0.007 | 0.007 | 0.005 | 0.006 | 0.007 | 0.006 | 0.006 | 0.007 |  |  |
| UMB | 0.009 | 0.008 | 0.005 | 0.008 | 0.007 | 0.007 | 0.004 | 0.005 | 0.006 | 0.005 | 0.005 | 0.006 | 0.005 |  |
| WGU | 0.009 | 0.009 | 0.006 | 0.008 | 0.008 | 0.008 | 0.005 | 0.006 | 0.007 | 0.006 | 0.006 | 0.007 | 0.006 | 0.007 |
