# Supplementary figures and images for: Mitochondrial and nuclear genetic analyses of the tropical black-lip rock oyster (Saccostrea echinata) reveals population subdivision and informs sustainable aquaculture development
Source: BMC Genomics. 2019 Sep 12;20:711. doi: 10.1186/s12864-019-6052-z (PMC6740020; doi:10.1186/s12864-019-6052-z)

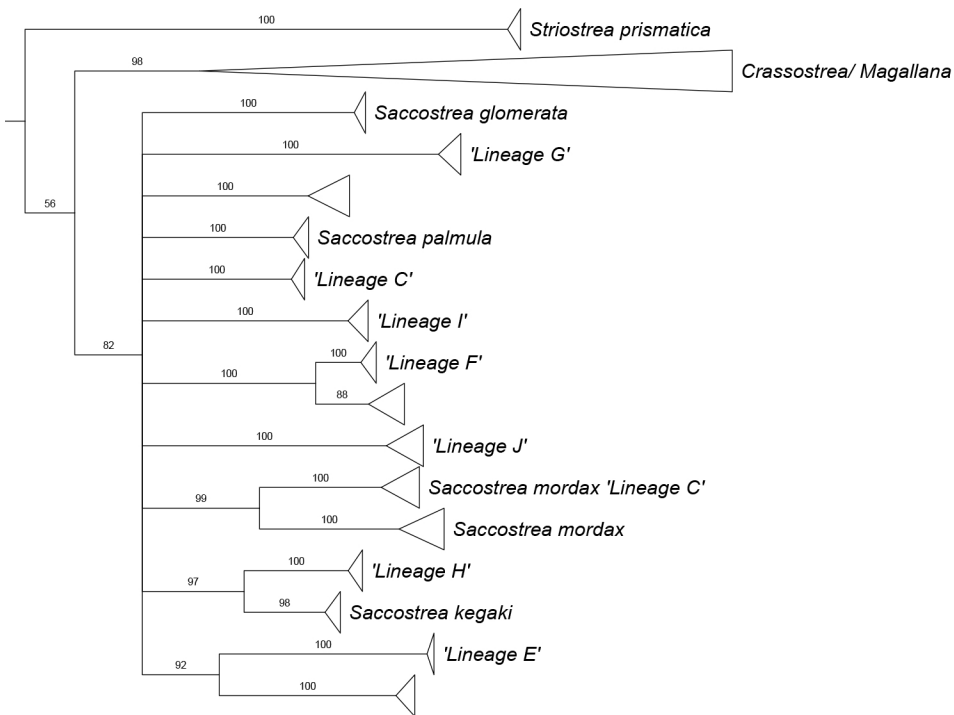

Supplement: Supplementary file 2 — Phylogenetic analysis of Saccostrea COI sequences. Bootstrap values are given on branches, and the scale bar indicates the number of substitutions per site. The clades containing Striostrea, Magallana and Crassostrea COI sequences are used as outgroups. Lineages have been designated (where possible) following Lam and Morton [86] and Sekino and Yamashita [32]. (PDF 386 kb) [file 12864_2019_6052_MOESM2_ESM.pdf]
