# Supplementary material for: Mitochondrial and nuclear genetic analyses of the tropical black-lip rock oyster (Saccostrea echinata) reveals population subdivision and informs sustainable aquaculture development
Source: BMC Genomics. 2019 Sep 12;20:711. doi: 10.1186/s12864-019-6052-z (PMC6740020; doi:10.1186/s12864-019-6052-z)
Supplement: Supplementary file 5 — This file includes a list of identifying numbers and sequences for the 31 candidate adaptive SNPs. (DOCX 14 kb) [file 12864_2019_6052_MOESM5_ESM.docx]

**Table A3** List of identifying numbers and sequences for the 31 candidate adaptive SNPs.

| **SNP ID** | **Sequence** |
| --- | --- |
| 927 | AATTCATTAATTATGTCAAATAAAAATTGGATATTTTAGTTTTATCTTTCATTTTTGCTATGATGGCAGAATATCCCAAACTCTCTTTAATTAAGGGGCACAATATGGGATCAAAATTATGGCTTGAACTATATAGAACT |
| 2350 | AATTCTTCCTTCTCCCAAAGCTTGAACTTGAATTGAAGCAAAGTTTTTAATTATAAGATCCTTCTTAATGATTTCTTTTTCTTCTCCATCATTCATTCTCTCTAACAAATGCATAACGTTACTTTCATTTTCATCAAACA |
| 10549 | AATTCAAGGTAGCAAAATGCCGCTTGGTAATGACATTACGTGACTCAGCTGATGACAGGGTTGCAGAAGAAGGGATTCAGACAAGGACAGGAAGAAAATGGTCAGCAAAAACAACAGTGGACCAAGCAGAGAGTATGTTA |
| 10895 | AATTCATTCCGTTGCCACTTTCAGAAAATTTCTCATCAATAACGACAACTTCCTTGTGTGTTTGACAATATTACCTGGTATTGATATGGGAAGTTTTTCTTAGACTTTGAAAAATCGTGTCTTTTGAATTTGCATCAAGT |
| 12806 | AATTCAACTTAATATAAAGCTACACTGTGGAAAGATTTCATATTTCTATTCATATCGAGATTATGCAACTTTTGCATTTAATGAGCATTTTTCTCTCTTTTTAGTTTGTTTGTTTATCTTTTAATTACTTTTTAAATCTT |
| 16542 | AATTCTTACGGCATTTTTTCATACTTCACAATGTATATGGATTGGATAAATTACGCACTTTTCAAGACAGCTTCAACGACAAAAAAAAGCATATGGGAAAAAATTCTGTAAATAATTATTTTAAAATTTACGATTGAAAT |
| 18035 | AATTCCAAATATAGCACAGTCCGCTTGACAAGGTTTCTCTCCAAGCAAAAACTTTTTCTGTCCTAAAACCACAAATAATAAACCAAATAAAGAACCTTATTCAAGCAAACTCAACTGCTATTTTCAAACTATTTATTTAC |
| 25829 | AATTCTAGTAAGATGAGTGTTTCGGAATTTTTCACCCCTTCATTGTGTCAGTCCTCTCAATATTGGACAGAATGTCATTTAAAAGCATCGAGTGTGAACAATTATGGAATGTCACAATTGGTGAAGCTCAAATTTGGTAT |
| 34199 | AATTCAAGCAAACTTTAAAAGATATGTGCTGCTTTGGTTCAACAAACCAAACAAAAACACCAAACACATATACTGCAAGAGTTGAAATGGACAAAAGTAATGGGAACTTGTCTGAAGCACAGGGCCTTTTGAAAGTTTCG |
| 35753 | AATTCGCGAAGAAACCGCGAAACAAATTGAAAAATAAACTCACCCAAATGAATCAATAGTTTAATACACAAATGACATTTATTATCTGGCAAATTGATTTATAAATGTAATAATAAAAGATATCGATTAAACATAGTAAA |
| 56518 | AATTCATATTCATACACAAAGTTCTAGTCAGAAAGGTCTCAAACTTTATCATATCAGGGAGTAAGACACTGAGAGACTTAAGCATAACACTAAAACACTATACTTATTTTAAAAACTCTTATTTACGCTTATTTGTTTTC |
| 73142 | AATTCTCTCTCTTTCTGAAAGTTAGAAGGACGTTCATCTGTTGTATATTTAGCAAAATGGAGATTTTTTTCTGATAAAAATTTATGTATGAATATCTCAGAACTTTACTCTTCTGACACAATACAAGGTTAAGTAAAACT |
| 77281 | AATTCATAAGAGTTTGTTTAATATATAATTTAGGACCGGCATAAAACAATATTCGGCACAGAATAACCCTGTGTGCAGGTTGCTTGGAAAAATTTGAATCCAAACTTAACATACTTTAAATACTTCGAAAGAGAAAAAAT |
| 82137 | AATTCGTCTATTAACCTGATAAAATTTCTAGAAGACTACTCCACATTGATCATTAATTACGGTGAAAGCTAAGTGCATCTCCTTTTAATTATTGCAATGAGAAGAGGGATTTTTTAGTGAGACATTCTATTGACAACGGC |
| 82504 | AATTCTATCATCAAAAATTAAATATAGCAAATATATTGTGAAAGTCTGGGGAAAAACCACTTTAAGCTTAAAATTAACACTATTGTAGTAATATCTTTTAAAAAATACATCATAGACCCCAACTTTTAATTTTCTTTTCT |
| 91474 | AATTCAAGGGTGAGGGATGATCAAATTTTGTGCGAAAATGCATTGATTTTTTGAAATAAGAGAAATGGACCCATTATTGATTATGATTTTACTTGATGTATGTAAAGTTATGAAAGAGAATAAAAATGGCTTAAGTACAT |
| 95664 | AATTCACAACGTGTTAAATTCGCCCTCTTCCTAATGAGGTGATAATAACACGGAGGTGAAAATATCTCTGTATAAAGTACAGTGTATATGTACTATTTTTCTTTGTTGATTTTATTAAAGTCTTAATTTCTCTCGGAATA |
| 103610 | AATTCGTAGTTCCAATAATTTCCAGATAAAATAAGTTTTTAATTATATTTTCGTGTGAAATGCTATTGCAATATATAAGTCGTGATATTATACAGAAAATTTGAGAAAATATGAAGTTCAAATCTGTACCAATGCACCTT |
| 109230 | AATTCAATGACTTATGGTTTCATTGGTCAGTGCCTAACGCTGAATCAGATTCTCTATATAGTTTGTTACTATTATTTTTTACCTTGTTGTTCAATATGGTTATTTATTTAACTATGTGCTGATAATAATAAACACAATTT |
| 120081 | AATTCAAAAATATTTTTTGATAGGTCCTAAATGGGCTTTCGTACTTTTCTGTGTTAGCATAAAATAATTATTGACTTGAACAATGATTGATCCCGTAAACCATTTCCAAATTGATTTTGCTTAATGTAAGACACAAAAAT |
| 121863 | AATTCCCTTAACTATACATTATTTTGTATAGTTGTATATATTTTGTAATTGTTAACCATAACAAAAGTACAAGAATGTGTTAAGCAATTAAAGGGTGTAAATTGGACAGTCCTTAAATAAGATTTGGAATATATAATATT |
| 124826 | AATTCAACCACACGGTGAACTAGCTTAATTTTTCGATGATGTACAATTACCCTCTAAACCACGGTGAACTGACACGAGCTCATAGGTCACAAAGTGCACATTAATATAAGCCTAAAAATATCAATGTAGGTTAATGTTTA |
| 125788 | AATTCCGGAATCAATCTAAAAACTATTCAGCGCTAAAGACGATTCTTTTTACATATAAACATATCTTTAATATGAGTTCTTCGGCAATTTATGTAAAGAAACACAGTTGTCACATATATTCTCATTTTCATCTTCTTGTG |
| 126470 | AATTCGGAGTTTTTGTTTCCTAGTTAATTTTGGCATTGAACTCTCAAAAATAAAAATTGTATATCCTCTGAATACAAGTGGTACGCAAAGATGATTTTAAAAAGGTAGCATTTAATCAATGATATAATTGGCAAATCAAA |
| 131848 | AATTCCACAATTTGGGAAGAAGTACTCTTTCTTATCATAACAATGCACTACCGTAACGTTTTTCTGGTATATGCTCAAGAGCAGAGAGGAAGATTTTCAAAGATTAAATGCATTTTCACTTTATGATCAATAGAGCCCCG |
| 135461 | AATTCTGGGAAGGTTTCATACAAAAGTTGGAAGGACAAATCTAATTCACATTATCTACTGTATACCTCAGTAATTAACACATTCTCAATGCTTTCTGATGACATTTTAAGAATTATCAATAGGTTTATGTAGCTAGTTTT |
| 178247 | AATTCTCGTATGTTAAGGTCAAAATCTAGTAAATGAATGAGGCCTACAGATTTCTAAAATTTGAGAAATAAATTCCTTTAATAATGCTTTATAACCGTAGCTTGGTCAAATTAGGTGTATCGGGGCATACTTTGTAGGTA |
| 260142 | AATTCTACTTATATCACTATCAATTCTGAAAGGAAAAAAAAGTGTTTACATATGAACGTGAAAATTGTCGTGAAAAAAGTATAGTTATAGTTAGGGTGAGTTAACCACGCATCGGACACATACCTTGCTTCGGACAACTT |
| 350690 | AATTCTATGGTCGTTATATAATGACCTTGTTAGCAAATATAACCAAACATAACCTACCATTTGGTCGTATTTCGGCTATTGTGTTTCATATCGATTGTTAGGACTTTGTATATATACTAAATTTGACTACAGATTGTTCC |
| 376479 | AATTCAAATTTTCTAAAAGTTCCTTGGAGTTCTCAAGTATCCACAACTGATTTACACCACTTCTCAAATACTGTTGTTAACTACTCCTGAAGTTTCTCACTCACTGCTGTAAATATTTCAGTAACATTTACTGAAACCCG |
| 427506 | AATTCGAGATAGCAAGGGTTAAATATTTAACCAGGGTTGAAATAATGGGGGATTTTCAAAAAGTAGAAGAGCGTCTGGAGTTCCTTCCTTAACAGGGGGTTGAGGGGGGGATACCAAAATAACTGGGGAATTATGTCATT |
